# First description of plasmid-mediated quinolone resistance determinants and β-lactamase encoding genes in non-typhoidal *Salmonella* isolated from humans, one companion animal and food in Romania

**DOI:** 10.1186/s13099-015-0063-3

**Published:** 2015-06-26

**Authors:** Liora Colobatiu, Alexandra Tabaran, Mirela Flonta, Ovidiu Oniga, Simona Mirel, Marian Mihaiu

**Affiliations:** Faculty of Pharmacy, Iuliu Hatieganu University of Medicine and Pharmacy, Cluj-Napoca, Romania; Faculty of Veterinary Medicine, University of Agricultural Sciences and Veterinary Medicine, Cluj-Napoca, Romania; Infectious Diseases Hospital, Cluj-Napoca, Romania

**Keywords:** *Salmonella*, Antimicrobial resistance, Resistance mechanisms, Humans, Companion animal, Food, Genetic relatedness, Public health

## Abstract

**Background:**

Gastroenteritis attributable to *Salmonella enterica* and the continuous increase in antimicrobial resistance of this gut pathogen, which compromises the use of previously effective treatments, is of great concern for public health. This study was conducted in order to investigate the presence of plasmid-mediated quinolone resistance (PMQR) determinants and β-lactamase-encoding genes, in *S.enterica*, isolated from humans, one companion animal and food. Moreover, the study aimed to identify potential vehicles of transmission of resistant strains to humans, with focus on food products (meat).

**Methods:**

A total of 20 *S. enterica* isolates recovered from food (chicken and pork meat), one companion animal and humans (stool samples), were examined for their serotype, antimicrobial susceptibility and the presence of PMQR and β-lactamase-encoding genes. Moreover, the genetic relatedness of nine *Salmonella* Infantis and ten *Salmonella* Enteritidis isolates was analyzed by pulsed-field gel electrophoresis (PFGE).

**Results:**

Among all isolates, 15 (75%) were multidrug-resistant (MDR) and the majority of them proved to be resistant to nalidixic acid and fluoroquinolones (FQs) (ciprofloxacin and levofloxacin). Twelve isolates (60%) harboured at least one PMQR gene [*qnrA*, *qnrB*, *qnrS*, *aac* (*6′*)-*Ib*-*cr* or *qepA*] while seven isolates (35%) carried at least one β-lactamase-encoding gene (*bla*_TEM_, *bla*_PSE-1_, *bla*_SHV_ or *bla*_CTX-M_). Moreover, two or more PMQR or β-lactamase-encoding genes co-existed in a single *S.enterica* isolate. A number of nine *Salmonella* Infantis, as well as the majority of *Salmonella* Enteritidis isolates analyzed by PFGE proved to be closely related.

**Conclusions:**

The study demonstrated the co-existence of PMQR and β-lactamase-encoding genes among the *Salmonella* isolates recovered and confirmed that multiple mechanisms might be involved in the acquisition and spread of resistance determinants. The close genetic relatedness between the clinical and foodborne *S. enterica* isolates, suggested that chicken meat might be a possible cause of human salmonellosis in our country, during the study period. Results of this study might improve understanding of the antimicrobial resistance mechanisms and transmission dynamics of *Salmonella* spp. Here, we report for the first time the presence of PMQR and β-lactamase-encoding genes in *S. enterica* isolates, recovered from humans, one companion animal and food, in Romania.

## Background

Gastroenteritis attributable to *Salmonella enterica* represents a global public health burden [1]. The main reservoir of *S. enterica* is the intestinal tract of many animal species and isolates are recovered from food and animal products such as meat from livestock, milk and eggs [2]. In 2013, a total of 85,268 confirmed cases of human salmonellosis were reported in the European Union (EU), among which, a number of 1,404 cases being reported in Romania [3].

Most of *Salmonella* infections are self-limiting, however, antimicrobial treatment is required, especially for invasive infections or severe diarrhoea. FQs and extended-spectrum β-lactams (especially extended-spectrum cephalosporins) are often the antimicrobials of choice for treating salmonellosis. Normally, FQs are not recommended in the treatment of infections in paediatric patients, because of their potential to cause arthropathy. However, FQs are still considered one of the last treatment options for life-threatening *Salmonella* infections, caused by MDR isolates [4].

Massive usage of antimicrobial agents in livestock production and also in human disease, increased and favored the survival of MDR pathogens and resistant *Salmonella* isolates recovered from different sources have been frequently reported worldwide [5–14].

The increasing prevalence of MDR among *Salmonella* spp., not only against the first-line antimicrobials such as ampicillin, chloramphenicol and trimethoprim/sulfamethoxazole, but also, against antimicrobial agents classified by the World Health Organisation (WHO) as critically important for human medicine, such as FQs and third generation cephalosporins (which have been extensively used in human and veterinary medicine), is an important emerging public health problem [15, 16].

The production of β-lactamases is the primary cause of resistance to β-lactams in Gram-negative bacteria and more than 340 β-lactamases have been described and classified. The extended spectrum β-lactamases (ESBLs) belong to class A and many of these enzymes are encoded by genes such as *bla*_TEM_, *bla*_PSE_, *bla*_PER_, *bla*_SHV_, *bla*_CTX-M_ and a few variants of *bla*_OXA_ [17]. Genes that encode β-lactamases can be located on the bacterial chromosome, plasmids, or transposable elements [17]. The most important high level of resistance to FQs is due to mutations in one or more of the genes that encode the primary and secondary targets of these drugs, the type II topoisomerases (*gyrA*, *gyrB*, *parC* and *parE*) [8, 11].

Various genes encoding different resistance mechanisms and located on mobile genetic elements, can also decrease susceptibility to quinolones (Qs) and FQs. These are often encoded on plasmids and known as PMQR genes [18].

Several PMQR mechanisms have been described: the determinant Qnr, which includes genes such as *qnrA*, *qnrB*, *qnrS*, with several variants each, and *qnrC* and *qnrD*, that increase resistance to both nalidixic acid and FQs [19, 20]; the cr variant of the common aminoqlycoside acetyltransferase aac(6′)-Ib, which is capable of reducing the activity of certain FQs [21]; the *QepA* determinant, an efflux pump that confers decreased susceptibility to hydrophilic FQs; and the multi-resistance efflux pump *OqxAB*, that is also able to confer resistance to nalidixic acid and ciprofloxacin, among other antimicrobial agents [22].

Food may act as a vector for the transfer of antimicrobial resistant bacteria and antimicrobial resistance genes to humans [23]. MDR bacteria can spread to humans either via the food supply (e.g., meat, fish, eggs and dairy products), via direct contact with animals or, more indirectly, through environmental pathways [24]. The consequences of antimicrobial resistance are particularly important when pathogens are resistant to antimicrobials that are critically important in the treatment of human disease [16].

Few new antimicrobial drugs are now in development, mainly represented by Qs (nemonoxacin), FQs such as avarofloxacin, zabofloxacin, finofloxacin or delafloxacin, tetracyclines (omadacyline, eravacycline), as well as a few new β-lactamase inhibitors (avibactam, relebactam) [25]. Therefore, considering that the success rate for drug development is low (at best, only 1 in 5 candidates that enter human testing will be approved for patients), maintaining the efficacy of the currently available antimicrobials is considered to be extremely important [25]. Knowledge regarding mechanisms of resistance and the path of resistant strains, is necessary in order to understand the conditions where resistance is selected and persists.

Until now, in Romania, no study has focused on the occurrence of PMQR determinants and of β-lactamase-encoding genes among *S. enterica* isolates recovered from food, companion animals and humans.

Therefore, this study was conducted in order to investigate the presence of PMQR determinants and β-lactamase-encoding genes in *S.enterica* isolated from humans, one companion animal and food. Moreover, the study aimed to identify potential vehicles of transmission of such strains to humans, with focus on food products (especially meat).

## Results

### Serotype distribution

Three different serotypes were identified among the recovered *S. enterica* isolates. The isolates consisted of ten isolates from stool samples of in-patients with diarrhea, nine food isolates (seven chicken meat and two pork meat) and one companion animal isolate, recovered from dog stool. *Salmonella* Enteritidis was the serotype most frequently detected (ten isolates, mainly recovered from human stool), followed by *Salmonella* Infantis (nine isolates, predominantly recovered from meat samples) and *Salmonella* Derby (one isolate) (Table 1).

**Table 1:** Phenotypic and genotypic features of the *Salmonella* isolates

No.	Serovar	Source	MIC (μg/mL)														PMQR/β-lactamase genes
			NAL	CIP	LEV	AMP	PIP	CTX	CAZ	GEN	AMK	TOB	NIT	SMX	TMT	TET	
1.	*S*. Infantis	Meat-chicken	>64	1	2	≥32	≤16	0.25	1	<1	<1	<1	≥512	>1,024	<1	>64	*bla*_SHV_, *qnrS*, *qepA*
2.	*S*. Infantis	Meat-chicken	>64	1	4	≥32	≤16	4	1	<1	<1	<1	≥512	>1,024	≥32	>64	*bla*_PSE-1_, *bla*_CTX-M_, *qepA*
3.	*S*. Enteritidis	Meat-chicken	<4	0.03	1	8	≤16	0.12	0.25	<1	<1	<1	≤32	64	<1	2	–
4.	*S*. Infantis	Meat-chicken	>64	2	4	8	≤16	0.25	1	<1	<1	<1	≤32	>1,024	≥32	>64	*qnrA*, *aac*(*6′*)-*Ib*-*cr*
5.	*S*. Infantis	Meat-pork	>64	0.5	4	8	≤16	0.25	0.5	<1	<1	<1	≤32	>1,024	<1	>64	*qnrB*, *qepA*
6.	*S*. Infantis	Meat-chicken	>64	0.5	4	8	≤16	0.25	1	<1	<1	<1	≤32	>1,024	≥32	>64	–
7.	*S*. Infantis	Meat-chicken	>64	0.5	2	4	≤16	0.12	0.5	<1	<1	<1	≥512	>1,024	<1	>64	*qnrA*, *aac*(*6′*)-*Ib*-*cr*
8.	*S*. Derby	Meat-pork	>64	1	2	2	≤16	0.25	1	<1	<1	<1	≤32	>1,024	<1	>64	*qnrA*, *qnrB*
9.	*S*. Infantis	Meat-chicken	>64	1	4	≥32	≥128	4	1	≤2	≤2	≤2	≥512	>1,024	≥32	2	*bla*_PSE-1_, *bla*_CTX-M_, *qnrS*
10.	*S*. Enteritidis	Human-stool	>64	1	2	8	≤16	0.25	0.5	<1	<1	<1	≥512	>1,024	<1	2	*qnrB*, *qnrS*, *aac*(*6′*)-*Ib*-*cr*
11.	*S*. Enteritidis	Human-stool	<4	0.03	1	4	≤16	0.12	0.5	<1	<1	<1	≤32	64	<1	2	–
12.	*S*. Enteritidis	Human-stool	<4	0.5	2	4	≤16	0.12	1	<1	<1	<1	≤32	64	<1	2	–
13.	*S*. Enteritidis	Human-stool	<4	1	2	≥32	≤16	0.25	1	<1	<1	<1	≤32	64	<1	2	*bla*_TEM_
14.	*S*. Enteritidis	Human-stool	>64	1	4	8	≤16	0.25	0.5	<1	<1	<1	≤32	64	<1	2	–
15.	*S*. Enteritidis	Human-stool	>64	2	4	≥32	≤16	0.25	0.5	<1	<1	<1	≥512	64	<1	2	*bla*_TEM_, *bla*_PSE-1_, *bla*_SHV_, *qnrA*, *aac*(*6′*)-*Ib*-*cr*
16.	*S*. Enteritidis	Human-stool	<4	0.03	1	2	≤16	0.12	1	<1	<1	<1	≤32	64	<1	2	–
17.	*S*. Enteritidis	Human-stool	<4	0.03	1	2	≤16	0.12	1	<1	<1	<1	≤32	64	≥32	2	–
18.	*S*. Enteritidis	Human-stool	>64	1	4	4	≤16	0.12	1	<1	<1	<1	≥512	>1,024	<1	2	*qnrA*
19.	*S*. Infantis	Human-stool	>64	1	4	≥32	≥128	4	1	≤2	≤2	≤2	≥512	64	<1	2	*bla*_SHV_, *qnrA*, *qnrS*, *aac*(*6′*)-*Ib*-*cr*
20.	*S*. Infantis	Dog-stool	>64	1	2	≥32	≥128	4	1	≤2	≤2	≤2	≥512	64	<1	2	*bla*_PSE-1_, *bla*_CTX-M_, *bla*_TEM_, *qnrB*, *qnrS*, *qepA*

*NAL* nalidixic acid, *CIP* ciprofloxacin, *LEV* levofloxacin, *AMP* ampicillin, *PIP* piperacillin, *CTX* cefotaxime, *CAZ* ceftazidime, *GEN* gentamicin, *AMK* amikacin, *TOB* tobramycin, *NIT* nitrofurantoin, *SMX* sulfamethoxazole, *TMT* trimethoprim, *TET* tetracycline, *PMQR* plasmid-mediated quinolone resistance, – no genes identified; *MIC* minimum inhibitory concentration.

### Antimicrobial susceptibility

In total, 14 (70%) of 20 isolates were resistant to nalidixic acid (MIC >64 μg/mL), 12 (60%) were resistant to ciprofloxacin (MIC 1–2 μg/mL), four isolates (20%) had reduced susceptibility to ciprofloxacin (MIC 0.5 μg/mL) and 16 isolates (80%) proved to be resistant to levofloxacin (MIC ≥2 μg/mL (Table 1).

Moreover, based on the β-lactams tested, seven isolates (35%) were resistant to ampicillin (MIC ≥32 μg/mL), 4 (20%) isolates were resistant to cefotaxime (MIC 4 μg/mL), 3 (15%) to piperacillin (MIC ≥128 μg/mL), while all isolates proved to be susceptible to ceftazidime (MIC 0.5–1 μg/mL) (Table 1).

A significant number of isolates were resistant to sulfamethoxazole (ten isolates, 50%; MIC >1,024 μg/mL) and nitrofurantoin (nine isolates, 45%; MIC >512 μg/mL).

A percentage of 15–35% of the isolates were resistant to other commonly used antimicrobials such as gentamicin, amikacin and tobramycin (three isolates, 15%; MIC 2 μg/mL), as well as trimethoprim (five isolates, 25%; MIC >32 μg/mL) and tetracycline (seven isolates, 20%; MIC >64 μg/mL).

### The presence of PMQR and β-lactamase-encoding genes

The results regarding the presence of PMQR and β-lactamase encoding genes are presented in Table 1.

#### Prevalence of PMQR genes

Out of 20 *Salmonella* isolates, 12 (60%) harboured at least one PMQR gene, the *qnr* genes (*qnrA* and *qnrS*) as well as *aac*(*6′*)-*Ib*-*cr* gene being the most prevalent ones detected. A number of 6 (30%) isolates contained *qnrA*, while 5 (25%) isolates contained the *qnrS* or *aac*(*6′*)-*Ib*-*cr* genes.

Of the eleven isolates containing a *qnr* gene, 6 (30%) contained *qnrA* (three isolates from human stool samples, one isolate from pork meat and two isolates from chicken meat), 4 (20%) isolates harbored *qnrB* (two isolates from pork meat, one from human stool and the isolate from dog stool), while *qnrS* gene was detected in 5 (25%) isolates (two from chicken meat and three from human stool).

*QepA* gene was also detected in 4 (20%) isolates (three from chicken meat and one from dog stool).

All PMQR-bearing isolates were resistant to nalidixic acid and levofloxacin and were also resistant or had reduced susceptibility to ciprofloxacin.

#### Occurrence of β-lactamase-encoding genes

Of the 20 *Salmonella* isolates, 7 (35%) carried at least one β-lactamase-encoding gene. Among these, 4 (20%) isolates carried *bla*_PSE-1_ gene and 3 (15%) isolates carried *bla*_SHV_, *bla*_TEM_ and *bla*_CTX-M_ genes. The *bla*_OXA_ gene was not identified in any of the isolates.

Two chicken meat isolates harbored both *bla*_PSE-1_ and *bla*_CTX-M_ genes (isolate 2 and 9). One human isolate carried *bla*_PSE-1_, *bla*_TEM_ and *bla*_SHV_ (isolate 15), while the isolate from dog stool (isolate 20) carried *bla*_PSE-1_, *bla*_TEM_ as well as *bla*_CTX-M_.

Almost all isolates carrying β-lactamase-encoding genes were also PMQR genes containing isolates, with the exception of a single human isolate.

### Genetic relatedness of *Salmonella* isolates

After digestion by *Xba*I enzyme, the genetic relatedness of nine *Salmonella* Infantis and ten *Salmonella* Enteritidis isolates was evaluated by PFGE. All isolates were typable by applying this method.

**Figure 1:**
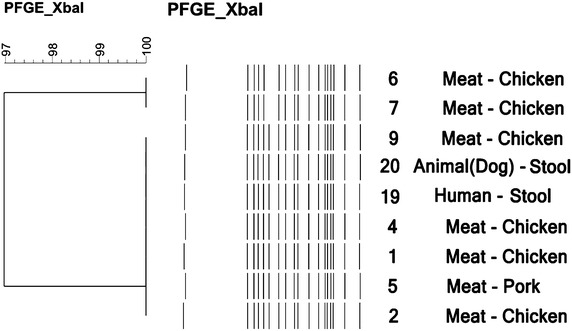
Dendrogram of PFGE patterns of genomic DNA digested by *Xba*I endonuclease in *Salmonella Infantis* isolates. The numbering of the strains from the dendogram is correlated to the numbering presented in Table 1.

Two *Xba*I-PFGE profiles showing 97% similarity have been identified among nine *Salmonella* Infantis isolates originating from different sources, including chicken and pork meat, as well as animal (dog) and human stool (Figure 1).

All nine *Salmonella* Infantis isolates were closely related, differing only by one band (Figure 1). One human isolate (isolate 19) and also the isolate from dog stool (isolate 20) displayed the same PFGE profile as four chicken meat isolates (isolates 1, 2, 4 and 9).

**Figure 2:**
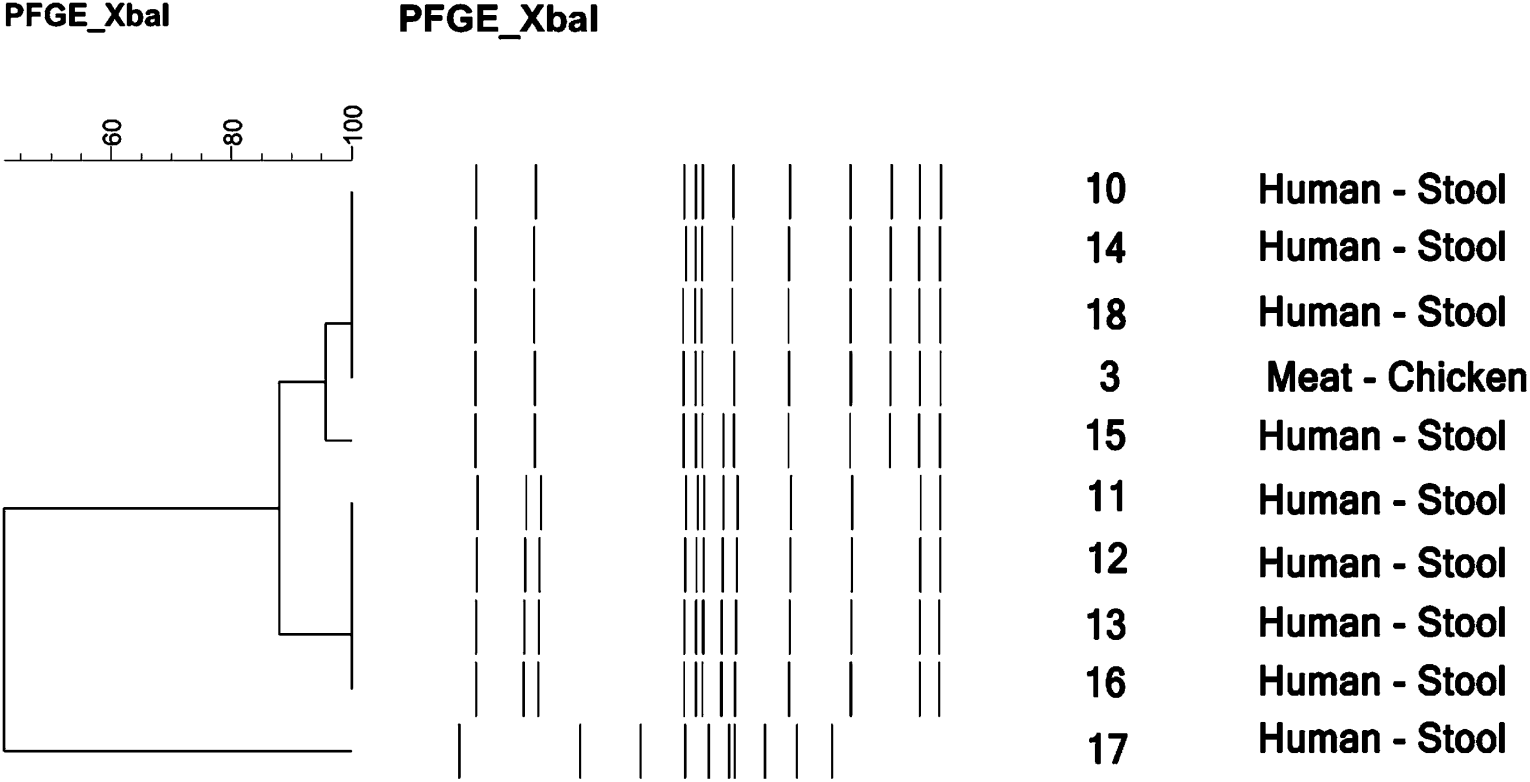
Dendrogram of PFGE patterns of genomic DNA digested by *Xba*I endonuclease in *Salmonella Enteritidis* isolates. The numbering of the strains from the dendogram is correlated to the numbering presented in Table 1.

*Salmonella* Enteritidis isolates recovered from humans and food (chicken meat) revealed four *Xba*I-PFGE profiles. One isolate from chicken meat (isolate 3) showed indistinguishable band pattern with that of three human isolates (isolates 10, 14 and 18) (Figure 2).

The majority of the isolates proved to be closely related, showing a similarity of 88%, with the exception of one human isolate (isolate 17) (Figure 2).

The dendrograms in Figures 1 and 2 demonstrate the banding patterns and the genetic relatedness of the isolates evaluated by PFGE.

## Discussion

In this study, 20 *S. enterica* isolates recovered from food (chicken and pork meat), one companion animal and humans (stool samples) were examined for their serotype, antimicrobial susceptibility and the presence of PMQR and β-lactamase-encoding genes. Moreover, the genetic relatedness of nine *Salmonella* Infantis and ten *Salmonella* Enteritidis isolates was analyzed by PFGE.

Three different serotypes were identified among the *Salmonella* isolates studied: *Salmonella* Enteritidis, *Salmonella* Infantis and *Salmonella* Derby. *Salmonella* Enteritidis was also the serotype most frequently detected among the human isolates, this being an important serovar causing non-typhoidal gastroenteritis in humans, in Europe [26].

In 2013, Romania reported a number of 1,404 cases of human salmonellosis (*Salmonella* Enteritidis being one of the two most commonly reported *Salmonella* serovars from cases of human infection, in Europe, in 2013) and one of the highest hospitalization proportions in the EU (80–95% of the cases being hospitalized). Interestingly, Romania is also one of the countries that reported the lowest notification rate of salmonellosis (6.5/100.000), which indicates that the surveillance system primarily captures the more severe cases [3].

In a previous study we reported that 70.87% of a total of 149 *Salmonella* isolates recovered from chicken and pork meat in Romania were serotyped as *S. enterica* subsp*. enterica* serovar Infantis. In this study, 45% of the isolates also belonged to *Salmonella* Infantis, being mostly recovered from food samples (chicken and pork meat) [5].

Among all isolates, 15 (75%) were MDR, exhibiting resistance to more than three antimicrobials, while three isolates (15%) proved to be susceptible to all the antimicrobials tested.

Of specific concern is the fact that the majority of the isolates proved to be resistant to Qs (nalidixic acid) and FQs (ciprofloxacin and levofloxacin). Qs and FQs are potent, broad-spectrum antimicrobial agents, commonly used in the treatment of infections in both humans and animals, being also used as prophylactic agents in food producing animals. Moreover FQs are included in the WHO’s list of critically important antimicrobials for human medicine [16]. In current human medical practice, the most commonly prescribed FQs are ciprofloxacin, levofloxacin and moxifloxacin. Despite prescribing guidelines recommending the prudent use of such antimicrobials, resistance continues to rise, as it was proven also in our study. Not only a high percentage of human isolates proved to be resistant to Qs and FQs, which was somehow expected, taking into consideration the extensive use of these agents in human medicine, but high resistance rates were also identified in the case of the meat isolates (Table 1). This could be explained by the fact that enrofloxacin is the second most used antimicrobial in veterinary practice in Romania (after tetracycline) and cross resistance among enrofloxacin, ciprofloxacin and other FQs has been noted [27].

In our country little information is available regarding antimicrobial susceptibility in *Salmonella* spp. isolated from food products, notification being mandatory only in poultry and for findings of *Salmonella* Enteritidis and *Salmonella* Typhimurium [28].

Resistance to β-lactams has also been observed in our study, 35% of the isolates being resistant to ampicillin and 15% to piperacillin. A number of four isolates (among which three from humans) proved to be resistant to cefotaxime, a third-generation cephalosporin.

The dramatic increase of the emergence and transmission of strains that are resistant to FQs and cephalosporins is considered to be an important public health concern.

Among the 20 isolates recovered, PMQR genes were found in 12 (60%). The prevalence and diversity of PMQR genes in this study is higher [*qnrA*, *qnrB*, *qnrS*, *aac* (*6′*)-*Ib*-*cr* and *qepA*] than reported for *Salmonella* isolates recovered from humans, animals and food in 13 European countries [29].

The most prevalent genes detected were represented by the *qnr* genes (*qnrA* and *qnrS*), as well as *aac*(*6′*)-*Ib*-*cr* gene. Although *qnrA* gene is more prevalent in *E. coli*, *Klebsiella* spp., or other *Enterobacteriaceae*, it has been rarely found in *Salmonella* in Europe [29, 30].

In general, PMQR mechanisms have been shown to reduce susceptibility to FQs and are considered to provide only a low level of resistance. However, their presence may stimulate mutations in genes encoding for DNA gyrase and toposiomerase IV [31].

Qnr determinants have been related to an enhanced facility to develop full resistance to Qs and FQs [32, 33], in agreement with the fact that decreased resistance levels to FQs favor the acquisition of full resistance to these agents [34]. Furthermore, two or more PMQR genes co-existing in a single isolate were frequently detected in our study (Table 1).

Five isolates harboured one of the *qnr* genes as well as the one that encodes aminoglycoside acetyltransferase, namely *aac*(*6′*)-*Ib*-*cr*, all being resistant to nalidixic acid, ciprofloxacin and levofloxacin (one isolate displayed intermediate susceptibility to ciprofloxacin). Although the expression of *aac*(*6′*)-*Ib*-*cr* alone is insufficient to cause complete ciprofloxacin resistance, when combined with the presence of *qnr*, its expression could result in ciprofloxacin resistance [35]. This might explain the high percentage of nalidixic acid and ciprofloxacin/levofloxacin resistant *Salmonella* isolates containing *qnr* and *aac*(*6′*)-*Ib*-*cr* genes.

Additional analysis would be necessary to reveal other possible resistance mechanisms such as point mutations in the quinolone resistance determining region (QRDR) or even the presence of new undetected PMQR genes.

Out of the 20 *Salmonella enterica* isolates recovered, 7 (35%) carried at least one β-lactamase-encoding gene (*bla*_TEM_, *bla*_PSE-1_, *bla*_SHV_ or *bla*_CTX-M_).

Three isolates also produced CTX-M type β-lactamases and proved to be resistant to cefotaxime. The CTX-M type β-lactamases have proved by far the most successful in disseminating in the clinical setting and have overall become the most prevalent ESBLs worldwide [36].

Of concern, we note that almost all isolates carrying a β-lactamase encoding gene also harboured PMQR genes.

Concern has been raised over the apparent correlation between the presence of PMQR genes [such as *qnr* and *aac*(*6′*)-*Ib*-*cr*] and plasmid-encoded ESBLs [9, 37].

PMQR genes are often carried on plasmids with other antimicrobial resistance genes, notably, genes encoding ESBLs. The carriage of PMQR genes, in association with other resistance genes, has been shown to influence the development of resistance to FQs, even in the absence of FQs drug pressure. Exposure to non-quinolone antimicrobials can favor persistance of PMQR determinants and consequent decreased susceptibility to FQs [9].

Recently, Vien et al. demonstrated that, after treatment for respiratory disease in Vietnam with non-quinolone antibiotics, there was significant enrichment for the carriage of PMQR genes in the gut flora of patients, even within days of treatment commencing. This was postulated to be due to co-selection of mobile genetic elements carrying multiple antimicrobial-resistance genes [38].

PFGE is considered to be a highly discriminative subtyping method in epidemiological investigation of many bacterial pathogens [39].

PFGE results in this study suggested that *Salmonella* isolates in each analysed serovar were closely related.

Among the *Salmonella* Infantis isolates, one human isolate (isolate 19) and also the isolate from dog stool (isolate 20) displayed the same PFGE profile as four chicken meat isolates (namely, isolate 1, 2, 4 and 9) (Figure 1). All of these isolates were highly resistant to antimicrobials and carried diverse PMQR and β-lactamase encoding genes. Among these, the human isolate 19, the isolate from dog stool (isolate 20) as well as one isolate from chicken meat (isolate 9), presented almost the same resistance pattern (NAL, CIP, LEV, AMP, PIP, CTX, GEN, AMK, TOB, NIT), with the exception of the chicken meat isolate, which proved to be resistant also to SMX and TET. Furthermore, these three isolates carried common PMQR and β-lactamase encoding genes, such as: *bla*_PSE-1_, *bla*_CTX-M_ and *qnrS* (Table 1). Isolates carrying the same antimicrobial resistance genes, and also belonging to the same PFGE profile, therefore being closely related, suggest the fact that clonal spread may be responsible for the dissemination of resistance determinants amongst the *Salmonella* Infantis isolates.

One *Salmonella* Enteritidis isolate from chicken meat (isolate 3) showed an identical band pattern with that of three *Salmonella* Enteritidis isolates recovered from human stool (isolates 10, 14 and 18) (Figure 2). Two of these human isolates (isolate 10 and 18) presented an identical resistance pattern (NAL, CIP, LEV, NIT, SMX) and both carried PMQR genes such as *qnrA*, *qnrB*, *qnrS* and *aac6′*(*Ib*)-*cr* (Table 1).

The close genetic relatedness identified between the chicken meat isolates and the *Salmonella* Infantis and Enteritidis human isolates indicate that chicken meat could be a possible source for infection with these serovars.

Food is generally considered to be the most important vector for the spread of resistance between humans and animals [40]. Foods contaminated with antimicrobial resistant bacteria could be a major threat to public health, as there is the distinct possibility that genes encoding antibiotic resistance determinants, that are carried on mobile genetic elements, may be transferred to other commensal or pathogenic bacteria of human clinical significance [41].

There is little information regarding the prevalence of antimicrobial resistance in *Salmonell*a spp., isolated from companion animals in many countries and no such related data has been published in Romania.

Taking into consideration the shared environment of humans and companion animals, transfer of resistant bacteria and even antimicrobial resistance genes between companion animals and humans is more likely to occur and has been indicated in some studies [42, 43].

## Conclusions

In conclusion, to our knowledge, this is the first study reporting the presence of PMQR and β-lactamase-encoding genes in *Salmonella* isolates recovered from humans, a companion animal and food and also describing the potential role of chicken meat in human non-typhoidal *Salmonella*, in Romania.

The study demonstrated the co-existence of PMQR and β-lactamase-encoding genes among the *Salmonella* isolates recovered and confirmed that multiple mechanisms might be involved in the acquisition and spread of resistance determinants.

The close genetic relatedness between the clinical and foodborne *Salmonella* isolates suggested that chicken meat might be a possible cause of human salmonellosis in our country, during the study period.

Admittedly, due to the limited number of isolates analysed, we are aware of the fact that the inclusion of more strains, especially isolated from different food sources, would be recommended in future studies, in order to accurately reflect the dissemination of MDR *Salmonella* isolates from food to humans. Moreover, further studies are needed to estimate the load of non-thypoidal *Salmonella* clinical cases, attributable to different identified food-originating serovars and to estimate the risk of the consumption of foods contaminated with MDR *Salmonella* carrying antimicrobial resistance determinants.

Results of this study might improve understanding of the antimicrobial resistance mechanisms and transmission dynamics of *Salmonella* isolates and highlight the importance of serious monitoring, thus aiding in stimulating the implementation of a continuous, integrated surveillance program at national level.

## Methods

*Salmonella enterica* isolates obtained in Romania from different sources, between March and August 2014, were investigated by pheno-and genotyping methods. The repertoire of antimicrobial resistance genes was determined by PCR.

### *Salmonella* isolates

A total of 20 geographically representative *S. enterica* isolates were recovered from humans (10 isolates from stool samples of in-patients with diarrhea), food isolates (seven chicken meat and two pork meat) and one companion animal (dog stool).

The majority of the isolates were isolated independently, between March and August 2014, at different points in time, from separate food samples or human clinical cases, respectively. Three isolates (one from chicken meat, one from a stool sample and the isolate from dog stool) were epidemiologically linked.

*Salmonella* spp., were isolated by previously homogenizing the samples (25 g) in buffered peptone water (225 mL) with a laboratory blender (Stomacher 400, England), for approximately 2 min. After incubation for 18–24 h at 37°C, 0.1 mL was inoculated in 10 mL Rappaport–Vassiliadis (RV) green broth (LabM Limited, England) and incubated for 18–24 h at 42°C. Another 1 mL from the culture obtained was inoculated into 10 mL of selenite cysteine (SC) broth (LabM Limited, England) and incubated at 37°C for 18–24 h. From both enrichment broths obtained, 1 mL was streaked onto brilliant green-phenol red-lactose-sucrose (BPLS) agar (Merck, Germany) and xylose lysine deoxycholate (XLD) agar (Oxoid, England). Following the incubation at 37°C for 24 h, presumptive *Salmonella* colonies were characterized by their biochemical properties through slide agglutination, using standard protocols. The positive colonies where then identified as *Salmonella* by using the Sensititre Automated Microbiology System Aris 2X (Thermo Scientific, England) following the protocol stated by the producer.

All strains of *Salmonella* spp., were confirmed by PCR, following the protocol reported in a previous study [5].

### Serotyping

All isolates of *S. enterica* included in the study were serotyped by serological identification of somatic (O) and flagellar (H) antigens, with commercially available antisera-*Salmonella* Antisera test group (Denka Seiken Co., England), according to the Kauffman-White serotyping scheme.

### Antimicrobial susceptibility testing

MICs were determined by using the reference broth microdilution method, according to the guidelines recommended by the Clinical and Laboratory Standards Institute (CLSI) [44]. The following antimicrobials were tested: ampicillin (AMP), piperacillin (PIP), cefotaxime (CTX), ceftazidime (CAZ), nalidixic acid (NAL), ciprofloxacin (CIP), levofloxacin (LEV), gentamicin (GEN), amikacin (AMK), tobramycin (TOB), nitrofurantoin (NIT), sulfamethoxazole (SMX), trimethoprim (TMP) and tetracycline (TET). The control strain used was *E. coli* ATCC 25922.

The breakpoints for the interpretation of resistance and susceptibility were determined according to the interpretive standards provided by the CLSI [44].

### Detection of PMQR determinants and of β-lactamase-encoding genes

All isolates were investigated for the presence of *qnr* (*qnrA*, *qnrB*, *qnrS*), *aac* (*6′*)-*Ib*-*cr* and *qepA* determinants and of *bla*_TEM_, *bla*_PSE-1_, *bla*_OXA_, *bla*_SHV_ and *bla*_CTX-M_ genes, through PCR amplification, using specific primers, previously reported [35, 45–51] (Table 2).

**Table 2:** Primers used for PCR

Primer	Sequence (5′–3′)	Target gene	Product (bp)	References
β-Lactamases				
TEM-F	CATTTCCGTGTCGCCCTTAT	*bla*_TEM_	793	[45]
TEM-R	TCCATAGTTGCCTGACTCCC			
PSE-1-F	AATGGCAATCAGCGCTTCCC	*bla*_PSE-1_	586	[46]
PSE-1-R	GGGGCTTGATGCTCACTACA			
OXA-F	ACCAGATTCAACTTTCAA	*bla*_OXA_	590	[47]
OXA-R	TCTTGGCTTTTATGCTTG			
SHV-F	TTATCTCCCTGTTAGCCACC	*bla*_SHV_	795	[48]
SHV-R	GATTTGCTGATTTCGCTCGG			
CTX-M-F	ACCGCGATATCGTTGGT	*bla*_CTX-M_	550	[49]
CTX-M-R	TTAGTGACCAGAATCAGCGG			
Plasmid-mediated quinolone resistance				
qnrA-F	ATTTCTCACGCCAGGATTTG	*qnrA*	516	[35]
qnrA-R	GATCGGCAAAGGTTAGGTCA			
qnrB-F	GATCGTGAAAGCCAGAAAGG	*qnrB*	476	[35]
qnrB-R	ACGATGCCTGGTAGTTGTCC			
qnrS-F	ACGACATTCGTCAACTGCAA	*qnrS*	417	[35]
qnrS-R	TAAATTGGCACCCTGTAGGC			
aac(6′)-Ib-cr-F	CCCGCTTTCTCGTAGCA	*aac*(*6′*)-*Ib*-*cr*	544	[50]
aac(6′)-Ib-cr-R	TTAGGCATCACTGCGTCTTC			
qepA-F	CGTGTTGCTGGAGTTCTTC	*qepA*	403	[51]
qepA-R	CTGCAGGTACTGCGTCATG			

#### DNA extraction

DNA was extracted from approximately 10–12 single colonies with the Isolate II DNA kit (Bioline, Germany), as specified by the manufacturer. DNA quantities and purities were assessed on a Nanodrop ND-1000 spectrophotometer analyzer (NanoDrop Technologies, Inc., Wilmington, DE, USA). Each sample was adjusted to a concentration of approximately 20 ng/µl in order to perform the PCR.

#### PCR protocol

Single PCR amplifications were performed in a 25 μL reaction mixture containing approximately 10 ng of template DNA and 2.5 pmol of each primer. The amplification protocol followed the steps accordingly: initial denaturation at 94°C for 3 min, followed by 30 cycles of amplification at 94°C for 45 s, annealing at 58°C for 30 s, and extension at 72°C for 30 s (except for the final cycle, which had an extension step of 5 min). The PCR products were analyzed on a 1.5% agarose gel.

### Genetic relatedness of *Salmonella* isolates

In order to determine the genetic relatedness of the *S. enterica* isolates, nine *Salmonella* Infantis and ten *Salmonella* Enteritidis isolates included in the study were analyzed by PFGE, according to PulseNet protocol [52] and previously described by Usein et al. [53].

Briefly, chromosomal DNA was digested with *Xba*I enzyme (Roche) and the DNA macrorestriction fragments were separated by electrophoresis, using a CHEF Mapper electrophoresis system (Bio-Rad, CA, USA), with pulse times of 2.16–63.8 s, during a 19 h run. *Salmonella* Braenderup H9812 was used as control strain.

The analyses of the gel images were carried out in BioNumerics v6.6.4 (Applied Maths, Kortrijk, Belgium). The comparisons were made by cluster analysis using Dice coefficient and dendrograms were generated using the unweighted-pair group method using average linkages (UPGMA) and a position tolerance of 1.5%.

Different profiles were assigned to *Xba*I-PFGE types according to differences in the band patterns, which were interpreted according to the criteria proposed by Tenover et al. [54].
